# Does a Low Glycaemic Index (GI) Diet Cost More during Pregnancy?

**DOI:** 10.3390/nu4111759

**Published:** 2012-11-15

**Authors:** Jane Cleary, Shelly Casey, Clare Hofsteede, Robert G. Moses, Marianna Milosavljevic, Jennie Brand-Miller

**Affiliations:** 1 Department of Nutrition, Wollongong Hospital, Wollongong, NSW 2500, Australia; Email: shelly.casey@sesiahs.health.nsw.gov.au (S.C.); marianna.milosavljevic@sesiahs.health.nsw.gov.au (M.M.); 2 School of Health Sciences, University of Wollongong, NSW 2500, Australia; Email: cmh067@uowmail.edu.au; 3 Illawarra Diabetes Services, P.O. Box W58, Wollongong, NSW 2500, Australia; Email: robert.moses@sesiahs.health.nsw.gov.au; 4 Boden Institute of Obesity, Nutrition, Exercise and Eating Disorders, University of Sydney, Sydney, NSW 2006, Australia; Email: jennie.brandmiller@sydney.edu.au

**Keywords:** GI, glycaemic index, pregnancy, food costs, food cost analysis, food economics, pricing

## Abstract

The aim of this study was to examine the monetary cost of dietary change among pregnant women before and after receiving low glycaemic index (GI) dietary advice. The pregnant women in this study were a subgroup of participants in the Pregnancy and Glycaemic Index Outcomes (PREGGIO) study. Twenty women from the low GI dietary advice group, who had completed their pregnancies, were randomly chosen. All these women had completed three day food records at 12–16 weeks and again around 36 weeks of gestation. Consumer food prices were applied to recorded dietary intake data. The mean ± SD GI of the diet reduced from 55.1 ± 4.3 to 51.6 ± 3.9 (*p* = 0.003). The daily cost of the diet (AUD) was 9.1 ± 2.7 at enrolment and 9.5 ± 2.1 prior to delivery was not significantly different (*p* = 0.52). There were also no significant differences in the daily energy intake (*p* = 0.2) or the daily cost per MJ (*p* = 0.16). Women were able to follow low GI dietary advice during pregnancy with no significant increase in the daily costs.

## 1. Introduction

Following a low glycaemic index (GI) diet can have health advantages [1]. There are reports of better glycaemic control [2], a reduction in total body fat, improved lipid profiles [3], and reduced risk of developing both diabetes [4] and cardiovascular disease [3]. A low GI diet has been demonstrated to reduce postprandial glucose levels, result in a vicarious higher fibre intake and an increased sense of satiety in healthy individuals [5].

In many instances lower GI food choices are more expensive than their more ubiquitous but higher GI equivalents. This has led to the perception that following a low GI diet is likely to be more expensive. The actual or perceived costs of food can sometimes be a disincentive to follow a diet [6,7]. A recent systematic literature review supports the view that food price affects food choices [8]. 

Pregnancy is an important time for maternal nutrition and a critical time for fetal nutrition. The mother provides the total environment for the developing and growing fetus and this can also influence long term health outcomes [9]. Elevated maternal glucose levels are associated with adverse pregnancy outcomes [10] and lowering the glucose improves these outcomes [11,12]. A low GI diet will result in babies that have a lower ponderal index and pregnancy outcomes with a lower proportion of large for gestational age (LGA) infants [13,14,15]. A low GI diet can also reduce the need for insulin use for women with gestational diabetes mellitus (GDM) without compromise of fetal outcomes [16]. 

To our knowledge, there has been no systematic examination of the costs of a low GI diet in general, and in pregnancy in particular. This study was designed to examine the costs of following a low GI diet in a group of otherwise healthy pregnant women. 

## 2. Experimental Section

The subjects and data for this study were obtained from the Pregnancy and Glycaemic Index Outcomes (PREGGIO) study. The PREGGIO study (ACTRN12610000174088) is an ongoing randomised controlled trial of a low GI diet compared with “healthy” eating in pregnancy. This is a translational study following on the positive pregnancy outcomes obtained from a low GI diet in a group of women who were supervised to a degree not really feasible for routine care [13]. Women for the PREGGIO study are being recruited at Wollongong Hospital with an expectation that about 700 will participate. Major inclusion criteria for women participating in the PREGGIO study are: <20 weeks gestation with a singleton pregnancy, age of ≥18 years, ability to read and understand a consent form and an ability to comply with the visit schedule. Major exclusion criteria include: known diabetes or previous gestational diabetes, women undertaking fad diets or with special dietary needs (e.g., coeliac disease, lactose intolerance), the presence of any serious medical conditions or using medications likely to alter body weight. 

Pregnant women are being enrolled at 12–16 weeks of gestation. Women in the low GI group were individually counseled to adopt diets that contain carbohydrates with a low GI and that also meet the nationally recommended nutritional intakes for pregnant women (*i.e.*, the Nutrient Reference Values for Australia and New Zealand) and the recommendations of the Australian Guide to Healthy Eating. They are provided with a booklet that outlines the low GI carbohydrate foods and what constitutes a serving and how many servings are required to meet their nutrient requirements for pregnancy. During these sessions, cheaper low GI products and recipes were usually discussed with those individuals who appeared concerned about costs.

All women are seen by a research dietitian and have a three day food record (two weekdays and one weekend day) taken a week after enrolment, before randomisation, and also at around 36 weeks of gestation. All women are instructed on how to use food records and to estimate their food intakes using standard serving sizes with the assistance of food models and measuring displays. Food records are reviewed with the research dietitian, when presented at initial and final visits to clarify food ingredients, presentation (e.g., Fresh, frozen), amounts, cooking methods, and brands. Food records are entered into a customized database incorporating the Australian food composition tables and published GI values, using the scale in which glucose equaled 100 (FoodWorks Professional, Version 6, 2009; Xyris Software, Brisbane, Australia). Additional GI data is obtained from an online database [17].

The study herein reported was initiated at the beginning of May 2011. At that time, 205 women had been recruited into the PREGGIO study, of whom 85 women randomised to the low GI diet (and 89 in the healthy diet group) had completed their pregnancies. 

For the purpose of this study, a random sample of 20 of the 85 subjects was selected using computer-generated random numbers. For a sample size of 20 there was an 80% chance of detecting an increase or decrease in cost of up to 25%. An increase in cost of more than 25% (equivalent to AUD 2.50) for a daily expenditure of AUD 10.00 was considered to be the minimum amount that would act as a disincentive. Food record data were analysed from the 20 women in the low GI group who had completed the PREGGIO study between February 2010 and May 2011. The adherence of women to the dietary advice was not assessed in the primary study and hence could not be considered in this sub-study.

A list of all foods reported in food records was constructed. The cost of the diet was estimated using an incident day technique, with the assumption that food costs on this day at a major supermarket chain (Woolworths) in a major regional city would provide a representation of average costs for the list of foods for the year. Prices of these food items were obtained from medium sized packages and were recorded in Australian dollars (AUD) as shown on Woolworths’ shelf-price labels expressed in weight or volume, such as in g, kg or mL or per single food (e.g., one medium apple). When recording food prices, the price of branded products were used when given or the least expensive product for items was recorded when only a food category was given. In our method, sale prices were not considered and the standard price was used. A separate list was compiled for foods which were specified as being purchased outside, or were not available in the supermarket, and the cost of these foods were obtained on the same day. These included vegetables (e.g., Carisma™ potatoes), bakery and fast food purchased in other food outlets. The average cost of out of season fruits and vegetables, not present in the shop on the day, were determined with the cooperation of the supermarket manager of fruit and vegetables and a consensus by the dietitians (JC, SC, NL, CH). Other general assumptions included all slices, cakes, muffins were priced in the bakery section. If a mixed meal or a recipe was included then these were disaggregated into ingredients and placed into food groups and priced separately. A list of assumptions were complied for a number of foods and recipes and were then placed in categories based on the Australian Dietary Guidelines [18]. The recorded prices of the foods obtained on the day were then transferred into the food records for each subject. The cost (per g or mL or each) was determined for each foods consumed. The cost of each day was determined by the sum of the cost of foods consumed in the day and then the average cost over the 3 days was calculated for each person. 

Basic descriptive and inferential statistical tests were performed using SPSS student version 19.0. (SPSS Inc., Chicago, IL). *t*-Tests were conducted to compare the sample to the population. The Wilcoxon rank-sum test was used to compare costs between the two groups before and after GI diet advice. A non-parametric test was utilised because we did not assume a normal distribution due to the small sample size. The level of significance was set at *p* < 0.05.

The PREGGIO and related sub-studies was approved by the South Eastern Sydney Illawarra Area Health Service and University of Wollongong Human Research Ethics Committee.

## 3. Results

The pre- and post-intervention three-day food records of all 20 randomly selected subjects were included in the final data analysis. By chance, all subjects were of a Caucasian background. No significant differences were found between the selected population when compared to that of the remainder (Table 1). 

**Table 1:** Characteristics of women in the low Glycaemic Index (GI) cost diet (*n* = 20) versus those in the Pregnancy and Glycaemic Index Outcomes (PREGGIO) study (*n* = 65).

	Low GI advice Costing Study (*n* = 20)	Low GI advice PREGGIO study (*n* = 65)	*p* Value ^a^
Age (years)	30.6 ± 5	28.7 ± 4	0.11
Ethnicity ^b^	100%	96%	
Pre-pregnancy Weight (kg)	70.0 ± 14.1	70.7 ± 16.8	0.86
BMI (kg/m^2^)	25.8 ± 6.5	25.7 ± 6.1	0.71
Gravida	2.1 ± 1.0	2.4 ± 1.2	0.34
Parity	0.7 ± 0.7	0.8 ± 0.7	0.64

^a^ *p* Value from *t*-test; ^b^ % expresses the proportion of women with Caucasian ethnicity.

The gestational age at the time of the first diet history was 15.9 ± 2.1 weeks. The final food records were taken at 36 ± 3.2 weeks. The changes in GI, dietary costs and energy intake before and after advice are shown in Table 2.

**Table 2:** Glycaemic index (GI), average daily cost (AUD), cost per MJ (AUD/MJ) and daily energy intake (kJ) before initiating a low GI diet and at 36 weeks gestation. Values expressed as mean ± SD.

	Before GI advice	After 36 weeks	*p* Value
**Glycaemic IndexRange**	55.1 ± 4.346.4–64.5	51.6 ± 3.942.0–59	0.003 *
**Daily Cost of Diet (AUD) Range**	9.1 ± 2.74.8–15.7	9.5 ± 2.14.8–14.2	0.52
**Daily Cost per MJ (AUD/MJ)**	1.0 ± 0.3	1.1 ± 0.2	0.20
**Daily Energy intake (kJ)**	9254 ± 1661	8617 ± 1852	0.16

* *p* Value from *t*-test.

## 4. Discussion

This study examined the changes made in both GI and food costs following nutrition education regarding both the quality and type of carbohydrate for pregnant women. At the end of their pregnancies the low GI dietary intervention, had a significant decrease of GI from 55.1 to 51.6, which was similar to the result of a previous study by Moses et al in a comparable population [13]. The GI was found to have a mean of 55.1 at the start of the study, which is lower than the average dietary GI being reported in Australia as between 56 and 58 [19]. This comparatively lower initial GI value may be attributed to an increase in the consumption of dairy, fruit and wholegrain products, which has been identified as the dietary changes commonly made by pregnant women [20,21,22]. Miller *et al.* [23] also identified a change in GI with participants with type 2 diabetes after receiving nutrition education, with a substantial change in the consumption of whole fruit, non-fat dairy products and vegetable fat. The change in food cost based on individual food groups was not examined. Our results also suggest that it is possible to lower dietary GI without incurring additional cost to the pregnant women. While, to our knowledge, there has been no previous studies relating to the cost of changing to a low GI diet following low GI diet advice, the literature related to costs and food choices is by no means consistent. 

Cross sectional studies found higher energy diets cost less whereas lower energy diets cost more [8,24,25,26,27]. A market basket survey study by Jetter *et al.* [28] revealed healthier market baskets to be significantly more expensive, primarily due to higher costs of wholegrains, lean ground beef, and skinless poultry. Similarly, Drewnowski *et al.* [29] and other researchers have also revealed through cost analyses that diets rich in fat and sugar are more affordable than diets high in fruits and vegetables [25,26,27]. Many of these studies utilise cost per calorie (or mega joule) comparison which can cause a two thousand calorie diet of fruit and vegetables (low energy dense, high nutrient) to be more expensive than that composed of high fat, high sugar foods (energy dense, low nutrition). 

Our study is more in line with the findings of longitudinal intervention studies, where dietary advice and intervention has been provided to specific groups. Mitchell *et al.* [30] who examined the food costs in low fat diets of obese children found that there was no significant difference in food costs between the low fat diet and a control group. Moreover, Rydén *et al.* [31] compared a Mediterranean diet to a standard Swedish diet and found no significant differences in non-energy adjusted costs between diets. Other researchers who have conducted cost benefit analyses of nutrition education programs have also found a significant decrease in spending after receiving nutrition education and advice [32,33]. It has been suggested that the differences in findings, can be attributed to the longitudinal examination of the data, rather than cross sectional, suggesting that over time, different food choices and cooking methods may be adopted that allow for lower food costs [34]. In contrast, women in a UK study indicated that a low fat diet increased costs due to an increase of fruit and vegetables but attributed this to the purchase of more expensive organic foods. They also acknowledged that at least half of the participants stated that it wasn’t difficult to eat healthily [26]. 

The purchase and consumption of products branded specifically as “low GI” is a factor which must be considered when examining the cost of following low GI dietary advice. While low GI products are available in many natural food sources, products developed and labelled as low GI such as low GI, Hi-Fibre breads may provide an easy choice for those following a low GI diet, and may incur additional costs. This finding was not found with our study, and could possibly be attributed to the support and nutrition advice provided on how to purchase low GI foods without the added expense. Similar findings have also been found with participants attending a healthy eating program who saved significantly more when receiving nutrition education when compared to a group that did not receive nutrition education [32]. Raynor *et al.* [33] also found that after families had received nutrition education that total daily food cost did not change at 6 months and decreased significantly at 12 months. 

Limitations of our study must be considered when interpreting the findings. Three-day food records are susceptible to known biases. Our study also had to assume that all foods, unless specified otherwise, were purchased within the supermarket. Additionally, some foods such as out of season fruit and vegetables were unavailable on the day and had to be priced as an average price for that product. General assumptions were also made about recipes and the ingredients used in these recipes. As prices were recorded only on one day they may differ from original prices paid due to daily price fluctuations.

A supermarket cost analysis was conducted rather than using online supermarket websites, national retail/food price surveys, nutrient databases or shopping catalogues, to allow for the maximal variety of foods from the food records to be priced and to emulate the most realistic means of shopping amongst Australian women. A major supermarket in Wollongong was chosen as the location for the cost analysis as the price of a food basket in Wollongong has been identified to being close to that of the average price of a food basket across supermarkets located in five suburbs within the region [24]. However, prices obtained at Woolworths Wollongong may not necessarily be representative of food prices in other parts of Australia.

The participants of the PREGGIO study following the healthy rather than the low GI diet were not included in this study for cost analysis before and after dietary education, due to resource constraints. Although the GI did not significantly decrease in this group, we are unable to say whether food costs increased or decreased after seeing the dietitian for healthy eating education for pregnancy. However this is certainly an area for future research from the database of the major study.

## 5. Conclusions

The study addresses a gap which exists in the current literature surrounding the monetary cost of changing to a low GI diet. This study found low GI dietary advice combined with dietary counseling resulted in significantly lowered dietary GI, with no significant change in monetary cost among randomly chosen participants of the PREGGIO study. Considering the interplay between price and value of infant and maternal health as major factors influencing food choice and dietary compliance, this result may have a positive impact on the acceptability and application of low GI dietary advice for pregnant women. 

## References

[1] Wolever T., Jenkins D., Jenkins A., Josse R. (1991). The glycemic index: Methodology and clinical implications. Am. J. Clin. Nutr..

[2] Thomas D., Elliott E. (2009). Low glycaemic index, or low glycaemic load, diets for diabetes mellitus. Cochrane Database Syst. Rev..

[3] Thomas D., Elliott E., Baur L. (2007). Low glycaemic index or low glyceamic load diets for overweight and obesity. Cochrane Database Syst. Rev..

[4] Barclay A., Petocz P., McMillan-Price J., Flood V.M., Prvan T., Mitchell P., Brand-Miller J.C. (2008). Glycemic index, glycemic load and chronic disease risk-a meta-analysis of observational studies. Am. J. Clin. Nutr..

[5] Brand-Miller J.C., Stockman R., Atkinson F., Petocz P., Denyer O. (2009). Glycemic Index, postprandial glycemia and the shape of the curve in healthy subjects: Analysis of a database of more than 2000 foods. Am. J. Clin. Nutr..

[6] Glanz K., Basil M., Maibach E., Goldberg J., Snyder D. (1998). Why Americans eat what they do: Taste, nutrition, cost, convenience, and weight control concerns as influences on food consumption. J. Am. Diet. Assoc..

[7] Blaylock J., Smallwood D., Kassel K., Variyam J., Aldrich L. (1999). Economics, food choices, and nutrition. Food Policy.

[8] Lee J.A., Ralston A., Truby H. (2011). Influence of food cost on diet quality and risk factors for chronic disease: A systematic review. Nutr. Diet..

[9] Tzanetakou I.P., Mikhailidis D.P., Perrea D.N. (2011). Nutrition during pregnancy and the effect of carbohydrates on the offspring’s metabolic profile: In search of the “perfect maternal diet”. Open Cardiovasc. Med. J..

[10] The HAPO Study Cooperative Research Group (2008). Hyperglycemia and Adverse Pregnancy Outcomes. N. Engl. J. Med..

[11] Crowther C.A., Hiller J.E., Moss J.R., McPhee A.J., Jeffries W.S., Robinson J.S., Australian Carbohydrate Intolerance Study in Pregnant Women (ACHOIS) Trial Group (2005). Effect of treatment of gestational diabetes mellitus on pregnancy outcomes. N. Engl. J. Med..

[12] Landon M.B., Spong C.Y., Thom E., Carpenter M.W., Ramin S.M., Casey B., Wapner R.J., Varner M.W., Rouse D.J., Thorp J.M. (2009). A multicenter, randomized trial of treatment for mild gestational diabetes. N. Engl. J. Med..

[13] Moses R.G., Luebcke M., Davis W.S., Coleman K.J., Tapsell L.C., Petocz P., Brand-Miller J.C. (2006). Effect of a low-glycaemic-index diet during pregnancy on obstetric outcomes. Am. J. Clin. Nutr..

[14] Clapp J.F. (1998). Effect of dietary carbohydrate on the glucose and insulin response to mixed caloric intake and exercise in both nonpregnant and pregnant women. Diabetes Care.

[15] Smith N., McAuliffe F., Quinn K., Lonergan P., Evans A.C. (2009). Transient high glycaemic intake in the last trimester of pregnancy increases offspring birthweight and postnatal growth rate in sheep: A randomised control trial. Br. J. Obstet. Gynaecol..

[16] Moses R.G., Barker M., Winter M., Petocz P., Brand-Miller J.C. (2009). Can a low-glycemic index diet reduce the need for insulin in gestational diabetes mellitus? A randomized trial. Diabetes Care.

[17] Glycemic Index Database, University of Sydney. http://www.glycemicindex.com.

[18] Commonwealth Department of Health and Aged Care (1998). Australian Guide to Healthy Eating.

[19] Barclay A., Flood V., Brand-Miller J.C., Mitchell P. (2008). Validity of carbohydrate, glycaemic index and glycaemic load data obtained using a semi-quantitative food frequency questionnaire. Public Health Nutr..

[20] Hook E. (1978). Dietary cravings and aversions during pregnancy. Am. J. Clin. Nutr..

[21] Anderson A.S., Campbell D., Shepherd R. (1993). Nutrition knowledge, attitude to healthier eating and dietary intake in pregnant compared to non-pregnant women. J. Hum. Nutr. Diet..

[22] Anderson A.S. (2002). Pregnancy as a time for dietary change?. Proc. Nutr. Soc..

[23] Miller C.K., Davis-Gutshcall M., Mitchell D.C. (2009). Change in food choices following a glycemic load intervention in adults with type 2 diabetes. J. Am. Diet. Assoc..

[24] Williams P., Hull A., Kontos M. (2009). Trends in affordability of the Illawarra Healthy Food Basket 2000-2007. Nutr. Diet..

[25] Drewnowski A., Monsivais P., Maillot M., Darmon N. (2007). Low-energy-density diets are associated withhigher diet quality and higher diet costs in French adults. J. Am. Diet. Assoc..

[26] Cade J., Upmeier H., Calvert C., Greenwood D. (1999). Costs of a healthy diet: analysis from the UK Women’s Cohort Study. Public Health Nutr..

[27] Brimblecombe J.K., O’Dea K. (2009). The role of energy cost in food choices for an Aboriginal population in northern Australia. Med. J. Aust..

[28] Jetter K.M., Cassady D.L. (2006). The availability and cost of healthier food Alternatives. Am. J. Prev. Med..

[29] Drewnowski A., Darmon N., Briend A. (2004). Replacing fats and sweets with vegetables and fruits-a question of cost. Am. J. Public Health.

[30] Mitchell D.C., Shannon B., McKenzie J., Smiciklas-Wright H., Miller B.M., Tershakovec A.M. (2000). Lower fat diets for children did not increase food costs. J. Nutr. Educ..

[31] Rydén P., Sydner Y.M., Hagfors L. (2008). Counting the cost of healthy eating: A Swedish comparison of Mediterranean-style and ordinary diets. Int. J. Consum. Stud..

[32] Burney J., Haughton B. (2002). EFNEP: A nutrition education program that demonstrates cost-benefit. J. Am. Diet. Assoc..

[33] Raynor H.A., Kilanowski C.K., Esterlis I., Epstein L.H. (2002). A cost-analysis of adopting a healthful diet in a family-based obesity treatment program. J. Am. Diet. Assoc..

[34] Frazão E., Golan E. (2005). Commentary: Diets high in fruit and vegetables are more expensive than diets high in fats and sugars. Evid. Based Healthc. Public Health.

